# Effect of Fermented Products Produced by *Bacillus licheniformis* on the Growth Performance and Cecal Microbial Community of Broilers under Coccidial Challenge

**DOI:** 10.3390/ani11051245

**Published:** 2021-04-26

**Authors:** Yeong-Hsiang Cheng, Yi-Bing Horng, Wei-Jung Chen, Kuo-Feng Hua, Andrzej Dybus, Yu-Hsiang Yu

**Affiliations:** 1 Department of Biotechnology and Animal Science, National Ilan University, Yilan 26047, Taiwan; yhcheng@ems.niu.edu.tw (Y.-H.C.); j9551792000@msn.com (Y.-B.H.); wjchen@niu.edu.tw (W.-J.C.); kuofenghua@gmail.com (K.-F.H.); 2 Department of Genetics, West Pomeranian University of Technology, 70-310 Szczecin, Poland; andrzej.dybus@zut.edu.pl

**Keywords:** *Bacillus licheniformis*, broiler, coccidiosis, fermented product, microbial community

## Abstract

**Simple Summary:**

Coccidiosis is a severe parasitic disease of poultry caused by parasites of the genus *Eimeria*. *Eimeria* species infection disrupts the intestinal microbiota of broilers, thereby reducing gut health and growth performance. Continuous use of anti-coccidial drugs leads to the selection of drug-resistant strains of *Eimeria*. Therefore, developing substitutes for anti-coccidial drugs is an urgent, unmet need. Fermented products produced by *Bacillus licheniformis* containing probiotics and antimicrobial peptides can modulate the gut microbiota of broilers. However, little is known about the effect of fermented products produced by *B. licheniformis* on the health, growth, and gut microbial community of broilers exposed to coccidial challenge. In this study, the anti-coccidial and gut microbiota modulatory effect of fermented products produced by *B. licheniformis* on broilers was evaluated. Results showed that *Bacillus licheniformis*-fermented product supplementation improved average daily gain at 21 to 35 days of age and regulated the cecal microbial community of broilers exposed to coccidial challenge compared with coccidial challenge alone group.

**Abstract:**

This study investigated the effects of fermented products produced by *Bacillus licheniformis* (fermented products) on the growth performance and cecal microbial community in broilers exposed to coccidial challenge. A total of 108 one-day-old male broiler chicks (Ross 308) were randomly allotted to one of three treatments. Each treatment was distributed into six replicate cages with six birds each. The treatments consisted of a basal diet without treatment (NC), basal diet plus coccidial challenge (PC), and basal diet plus the coccidial challenge and 1 g/kg of fermented products (FP). The results indicated that FP increased the average daily gain of broilers at 21 to 35 days of age compared with the PC group (*p* < 0.05). The anti-coccidia index in the FP group was elevated compared with the PC group (*p* < 0.05). Principal coordinate analysis showed significant segregation in bacterial community composition in the cecal digesta among the groups. The genus *Lactobacillus* was more abundant in the cecal digesta of the FP group compared with the PC group (*p* < 0.05). There was a positive correlation between the abundance of the genus *Lactobacillus* in the cecal digesta and growth performance (body weight, average daily gain, and average feed intake). Furthermore, the abundance of the genus *Lactobacillus* in the cecal digesta was positively associated with the cecal short-chain fatty acid levels (formic acid, acetic acid, propionic acid, butyric acid, and isobutyric acid). These findings suggest that fermented products produced by *B. licheniformis* can ameliorate the average daily gain of broilers exposed to coccidial challenge. *B. licheniformis*-fermented product supplementation increases anti-coccidial activity and modulates gut microbiota composition by increasing beneficial microbes and decreasing harmful microbes in broilers under coccidial challenge.

## 1. Introduction

Coccidiosis is a severe enteric disease in poultry caused mainly by protozoa from the *Eimeria* genus. Coccidiosis costs the poultry industry about 3 billion US dollars annually worldwide due to high mortality, impaired growth, and high medical costs [1]. Anti-coccidial drugs have been widely used to control coccidiosis, but drug resistance of *Eimeria* species in chickens has become prevalent worldwide. Because of the disadvantages of current anti-coccidial drugs and vaccines [2], alternative strategies to prevent coccidiosis in broilers are an urgent and unmet need in the poultry industry. Several natural products, such as medicinal plants, herbal extracts, essential oils, organic acids, and probiotics, have been reported to have beneficial effects for the prevention of coccidiosis [3,4,5,6,7,8].

*Bacillus licheniformis*, a Gram-positive endospore-forming probiotic, has either been observed or isolated from the digestive tract of broilers [6]. Dietary supplementation with *B. licheniformis* ameliorates the growth performance and relieves necrotic enteritis caused by *Clostridium perfringens* in broilers [9,10,11]. However, the beneficial effects of *B. licheniformis* as probiotics in broilers infected with coccidian parasites are still limited. Only one study reported that *B. licheniformis* can improve body weight gain, intestinal lesion score, and fecal oocysts in broilers challenged with mixed coccidia infection [12]. The underlying mechanisms of how *B. licheniformis* prevents coccidiosis of broilers are still unclear.

The commercial feed additive of *B. licheniformis* is mainly produced by liquid-state fermentation and fermented products (*B. licheniformis* spores only, without functional metabolites) are then directly mixed into the diet. Compared with liquid-state fermentation, our previous study demonstrated that *B. licheniformis* can be produced by solid-state fermentation. Fermented products contain *B. licheniformis* spores and functional metabolites (antimicrobial cyclic lipopeptide) [13]. Furthermore, *B. licheniformis*-fermented products also display antimicrobial activity against *C. perfringens* and *Brachyspira hyodysenteriae* in vitro [13,14]. Dietary supplementation with fermented products produced by *B. licheniformis* can ameliorate growth performance, alleviate necrotic lesions, and improve intestinal morphology in broilers exposed to *C. perfringens* challenge [15,16].

In addition, fermented products also modulate intestinal bacterial composition in broilers [15]. It has been reported that *Eimeria tenella* infection induces perturbation of the cecal microbiota in different strains of broilers by elevating pathogenic bacteria abundance and reducing beneficial bacteria abundance [17,18]. Therefore, based on our previous studies, we hypothesize that fermented products might alleviate coccidiosis by normalizing the cecal microbiota composition of broilers.

The specific objectives of the present study were to investigate the effects of fermented products produced by *B. licheniformis* on growth performance and cecal microbial community in broilers exposed to coccidial challenge. The findings provide a basis for the use of *B. licheniformis*-fermented products as a possible method for preventing coccidia in poultry.

## 2. Materials and Methods

The Institutional Animal Care and Use Committee of National Ilan University reviewed and approved the animal protocol for the current study (IACUC, protocol number 107-12).

### 2.1. Experimental Design

The fermented products were produced in our previous study and the concentration of *B. licheniformis* and *B. licheniformis*-derived antimicrobial cyclic lipopeptide (surfactin) in fermented products were 5 × 10^11^ CFU/g and 10 mg/g, respectively [16]. A total of 108 one-day-old male broiler chicks (Ross 308) obtained from a local commercial hatchery with an average body weight of 43.96 ± 0.05 g were randomly allotted to one of three treatments in a completely randomized design. Each treatment was distributed into six replicate cages with six birds each. Broilers were reared in stainless-steel, temperature-controlled cages (190 cm × 50 cm × 35 cm).

The treatments consisted of (1) basal diet without treatment (NC), (2) basal diet plus coccidial challenge (PC), and (3) basal diet plus the coccidial challenge and 1 g/kg of fermented products (FP). The experimental diets were formulated to meet or exceed the requirements of birds according to National Research Council recommendations (Nutrient Requirements for Poultry, 1994, Table 1). In the FP group, the soybean meal in the basal diet was replaced with fermented products equally. No coccidiostats and antibiotics were included in the diets. The broilers were on the test diets from 1 to 35 days of age. The feeding program had two phases that spanned days 1–20 and days 21–35.

The birds were given drinking water and feed *ad libitum*. A 23 light: 1 dark photoperiod was applied for the first week and then a 20 light: 4 dark photoperiod was applied after the first week. Ambient temperature on days 1 to 3 was set at 33 °C and gradually reduced to 30 °C on days 4 to 7, 27 °C on days 8 to 14, and 24 °C on days 15 to 35.

Newcastle disease–infectious bronchitis vaccination programs were performed on days 4 and 14 by nose drop administration (multivalent vaccine containing live Newcastle disease virus, B1 type, B1 strain and live IB virus, Massachusetts and Connecticut serotypes, Zoetis, Parsippany, NJ, USA). The commercial coccidial vaccine (Coccidiosis Quadrivalent Vaccine for Chickens, Guangdong Wens Dahuanong Biotechnology, Guangdong, China) containing anti-coccidial-sensitive strains of *E. tenella*, *E. maxima*, *E. necatrix*, and *E. acervulina* is a live oocyst isolated from chickens. A dose of the vaccine (1×), as recommended by the manufacturer, contains approximately 1100 oocysts. To mimic the *Eimeria* species challenge, a 10× dose of the commercial coccidial vaccine (approximately 11,000 oocysts and dissolved in distilled water) was administered to broilers in challenged groups (PC and FP) by oral gavage on day 14, whereas birds in the unchallenged group (NC) were orally gavaged with distilled water. Body weight and feed intake on a pen basis were evaluated every week and every day, respectively. The growth performance (average body weight, average daily gain, average daily feed intake, and feed conversion ratio) was calculated from two feeding phases (days 1–20 and days 21–35). The mortality of broilers was monitored daily.

### 2.2. Evaluation of Anti-Coccidial Index

The anti-coccidial index (ACI) was calculated based on the following formula, ACI = [relative body weight gain (RBWG, %) + survival rate (SR, %)] − [lesion score index (LSI) + oocyst count index (OI)]. The RBWG and SR of all broilers were recorded from days 14 to 35. For LSI analysis, two broilers per replicate were selected at the end of the experiment (day 35) based on their cage’s average body weight and then euthanized using carbon dioxide inhalation. Both ceca from each broiler were freshly collected for macroscopic LS evaluation using the method established by a previous study [19]. Two broilers per replicate were chosen based on their cage’s average weight for OI analysis. Feces from each broiler were freshly collected daily from days 14 to 35 in an independent cage. After daily fecal sample collection, fresh feces from two broilers were weighed, pooled, suspended in water, and counted on McMaster egg-counting chambers (Vetlab Supplies, West Sussex, United Kingdom). Oocysts per gram of feces (OPG) were calculated from the average of 3 counts of each fecal sample. OI was calculated as follows: 100 × 0.4 × (oocyst counts per group)/oocyst counts for the coccidial challenge alone group.

### 2.3. 16S Ribosomal RNA Gene Sequencing and Data Processing

For microbiota analysis, two broilers per replicate were chosen at the end of the experiment (day 35) based on their cage’s average weight and then euthanized using carbon dioxide inhalation (birds chosen for microbiota analysis were identical to those for LSI analysis). Fresh digesta from the cecum of two broilers were sampled and pooled from each replicate. Three replicates (n = 3) were used for 16S ribosomal RNA gene sequencing. The total genomic DNA from cecal digesta was extracted and purified using a ZymoBIOMICS DNA Miniprep kit (Zymo Research, Irvine, CA, USA). Total DNA quantitative and qualitative analyses were measured by a Quantus Fluorometer (Promega, Madison, WI, USA) and agarose gel electrophoresis, respectively. The V3–V4 hypervariable region of the 16S rRNA gene from individual samples was amplified using 341F-805R primer (5′-CCTACGGGNGGCWGCAG-3′ and 5′-GACTACHVGGGTATCTAATCC-3′). The PCR amplicons were purified using a QIAquick Gel Extraction kit (QIAGEN, Germantown, MD, USA). Sequencing libraries were produced and sequenced at a read length of 300 nucleotides on a MiSeq platform (Illumina, San Diego, CA, USA). The sequence data were processed using the QIIME 2 software package (version 2017.4, GitHub, San Francisco, CA). High-quality reads were selected and all of the effective reads from all samples were clustered into operational taxonomic units (OTUs) based on 97% sequence similarity using UCHIME (version 4.2, GitHub) and mothur software (version 1.39.5, GitHub). Alpha diversity (richness and evenness) and phylogenetic assignment were accessed using QIIME 2 software (version 2017.4, GitHub) and naïve Bayesian classification method, respectively. The principal component analysis (PCA) and principal coordinate analysis (PCoA) based on the unweighted and weighted UniFrac distance matrices were used to visualize the difference of microbiota among groups using the R packages (version 3.5.0 and version 1.7.13, GitHub). The functions of all the OTUs were predicted by Kyoto Encyclopedia of Genes and Genomes (KEGG) databases using PICRUSt software (version 1.1.4, GitHub). Correlation analysis was performed using the Correlogram (version 0.84, GitHub).

### 2.4. Cecal Short-Chain Fatty Acid Measurement

For cecal short-chain fatty acid extraction, two broilers per replicate were chosen at the end of the experiment (day 35) based on their cage’s average weight and then euthanized using carbon dioxide inhalation (birds chosen for short-chain fatty acid analysis were identical to those for LSI and microbiota analysis). Fresh digesta from the cecum of two broilers were sampled and pooled from each replicate. Three replicates (n = 3) were used for the quantification of short-chain fatty acid. The short-chain fatty acids were analyzed by gas chromatography-mass spectrometry (Bruker GC-MS System, Burker Corp., Billerica, MA, USA). Briefly, cecal digesta was extracted with 10% isobutanol and homogenized. After centrifugation, the supernatant was isolated and mixed with NaOH and chloroform. The aqueous phase of the mixture was mixed with isobutanol, pyridine, and isobutyl chloroformate and sonicated. The mixture was then extracted with hexane. After centrifugation, the short-chain fatty acid contents in the supernatant were analyzed by gas chromatography-mass spectrometry. The separations were performed on low-bleed GC/MS columns (VF-5ms, 30 m × 0.25 mm; Agilent, Santa Clara, CA, USA) at a flow rate of 1.0 mL/min helium as a carrier gas. The electron energy was 70 eV. The oven temperature was held at 40 °C for 5 min, then ramped to 310 °C at a rate of 10 °C min^−1^. Injection volumes for all samples and standards were 2.0 L with a 10:1 split ratio. The cecal short-chain fatty acids measured were formic, acetic, propionic, butyric, and isobutyric acid.

### 2.5. Statistical Analysis

Replicates were considered to be the experimental units. Individual cages were defined as replicates for each determined parameter. The Student’s *t*-test (two-tailed) was used for intergroup comparison in SAS software (version 9.4, 2012; SAS Institute, Cary, NC, USA). *p* ≤ 0.05 was considered statistically significant. The PCoA analysis based on UniFrac distances coupled with standard multivariate statistics was assessed. The relationship between the dominant 10 genera, growth performance, and short-chain fatty acid levels was assessed using Pearson’s correlation coefficient (r).

## 3. Results

### 3.1. Effect of Fermented Products Produced by B. licheniformis on the Growth Performance, Anti-Coccidial Index, and Cecal Short-Chain Fatty Acid Levels of Broilers Exposed to Coccidial Challenge

No dead birds were observed over the experimental period. The effect of fermented products produced by *B. licheniformis* on the growth performance of broilers under coccidial challenge is described in Table 2. The PC group had a reduced body weight at 35 days of age compared with the NC group (*p* = 0.031). The PC group had a reduced average daily weight gain between day 21 and day 35 of age (*p* = 0.037) and this was also significant during the whole experimental period (day 1 to 35 of age) compared with the NC group (*p* = 0.031). Fermented product supplementation increased the average daily gain between day 21 and day 35 of age compared with the PC group (*p* = 0.05). The average daily feed intake was increased between day 21 and day 35 of age (*p* = 0.012 and *p* = 0.003) and the whole experiment period (*p* ≤ 0.001 and *p* = 0.006) in the PC and FP group compared with the NC group. PC and FP group had a worsened feed conversion ratio at 21 to 35 days of age (*p* = 0.014 and *p* = 0.023) and the whole experiment period (*p* = 0.011 and *p* = 0.02) compared with the NC group. PC group had a lower anti-coccidial index compared with the NC group (*p* ≤ 0.001). The anti-coccidial index was increased in the FP group compared with the PC group (*p* ≤ 0.001). The effect of fermented products on the cecal short-chain fatty acids of broilers at 35 days of age in response to coccidial challenge is described in Table 3. There were no significant differences between the NC and PC group in all short-chain fatty acid levels in cecal digesta. Fermented product supplementation could increase the propionic acid levels in the cecal digesta of broilers compared with the NC and PC group (*p* = 0.02 and *p* ≤ 0.001).

### 3.2. Effect of Fermented Products Produced by B. licheniformis on the Cecal Bacterial Community Composition of Broilers in Response to the Coccidial Challenge

**Figure 1:**
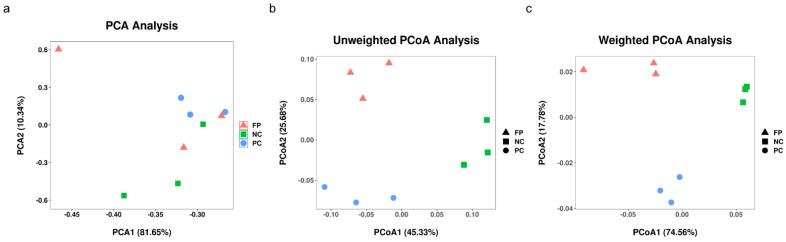
Advanced analysis of the bacterial communities of cecal digesta. (**a**) Principal component analysis of the cecal digesta of basal diet without treatment (NC), basal diet plus coccidial challenge (PC), and basal diet plus the coccidial challenge and 1 g/kg of *B. licheniformis*-fermented products (FP) (n = 3). Principal coordinate analysis of quantitative traits (unweighted UniFrac distances) (**b**) and qualitative traits (weighted UniFrac distances) (**c**) of the cecal bacterial communities from NC, PC, and BLFP (n = 3).

At 35 days of age, the averages of high-quality reads and OTUs from the cecal digesta of broilers fed only a basal diet, basal diet plus coccidial challenge, or basal diet plus coccidial challenge and fermented products were 71,381 and 5235, 72,941 and 6543, 73,972 and 6328, respectively. The PC group had an increased species richness (Chao1, *p* = 0.016 and Fisher alpha, *p* = 0.031) and species evenness (Shannon, *p* = 0.004 and Enspie, *p* = 0.002) levels in the cecal digesta of broilers compared with the NC group (Table 4). The species richness (Chao1, *p* = 0.033 and Fisher alpha, *p* = 0.045) and species evenness (Shannon, *P* = 0.016) was increased in the FP group in the cecal digesta of broilers compared with the NC group. There were no significant differences between the PC and FP groups in the species richness and species evenness in the cecal digesta of broilers (Table 4). The PCA result revealed that OTU composition among groups was not well-separated (Figure 1a). In contrast, unweighted PCoA (qualitative traits) and weighted PCoA (quantitative traits) exhibited significant segregation in bacterial community composition in the cecal digesta among the groups (Figure 1b,c).

### 3.3. Effects of Fermented Products Produced by B. licheniformis on the Cecal Bacterial Taxonomic Distribution in Broilers Exposed to Coccidial Challenge

The results of bacterial taxonomic assignment and ranking in the cecal digesta of broilers are shown in Table 5. At the phylum level, the abundance of the phyla *Firmicutes* (*p* = 0.015) and *Actinobacteria* (*p* = 0.01) was increased and the abundance of the phylum *Proteobacteria* (*p* = 0.009) was reduced in the PC group compared with the NC group. Fermented product supplementation could increase the abundance of the phylum *Proteobacteria* (*p* = 0.049) and decrease the abundance of the phylum *Actinobacteria* (*p* = 0.03) compared with the PC group. The abundance of the phylum *Firmicutes* (*p* = 0.025) was increased and the abundance of the phylum *Proteobacteria* (*p* = 0.021) was decreased in the FP group compared with the NC group. At the genus level, the abundance of the genera *Ruminococcus_torques_group* (*p* = 0.022), *Ruminiclostridium_9* (*p* = 0.001), *Butyricicoccus* (*p* ≤ 0.001), *Blautia* (*p* ≤ 0.001), *Eubacterium_hallii_group* (*p* = 0.016), and *Ruminiclostridium_5* (*p* = 0.004) were increased and the abundance of the genera *Lachnospiraceae_unclassified* (*p* ≤ 0.001), *Lactobacillus* (*p* = 0.026), and *Sellimonas* (*p* = 0.001) were decreased in the PC group compared with the NC group. Fermented product supplementation could increase the abundance of the genera *Lactobacillus* (*p* = 0.031) and *Blautia* (*p* = 0.009) and decrease the abundance of the genera *Ruminococcus_torques_group* (*p* = 0.003) and *Romboutsia* (*p* = 0.003) compared with the PC group. The abundance of the genera *Lactobacillus* (*p* = 0.045), *Ruminiclostridium_9* (*p* ≤ 0.001), *Butyricicoccus* (*p* ≤ 0.001), *Blautia* (*p* ≤ 0.001), *Eubacterium_hallii_group* (*p* = 0.001), and *Ruminiclostridium_5* (*p* = 0.005) were increased and the abundance of the genera *Lachnospiraceae_unclassified* (*p* = 0.032), *Ruminococcus_torques_group* (*p* = 0.019), *Sellimonas* (*p* = 0.015), and *Romboutsia* (*p* = 0.001) and were decreased in the FP group compared with the NC group.

**Figure 2:**
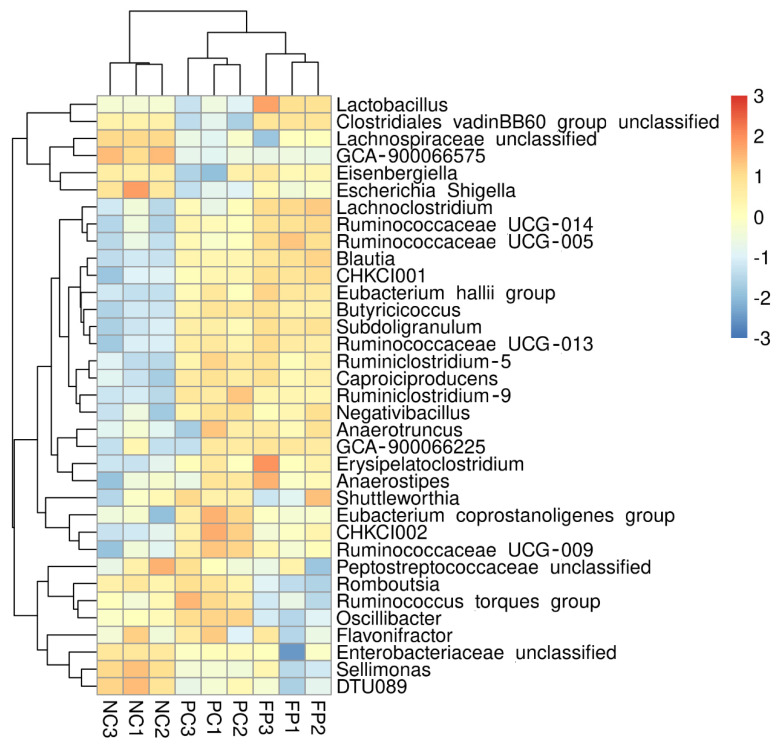
Taxonomic composition analysis of cecal digesta. A heatmap showing the dominant 35 genera (*y*-axis) across different treatment groups (*x*-axis, basal diet without treatment (NC), basal diet plus coccidial challenge (PC), and basal diet plus the coccidial challenge and 1 g/kg of *B. licheniformis*-fermented products (FP), n = 3).

Furthermore, similar microbial community clusters (coccidial oocysts-responsive bacterial clusters), such as genera *Ruminococcaceae_UCG_014*, *Blautia*, *CHKCI001*, *Caproiciproducens*, *Subdoligranulum*, *Ruminococcaceae_UCG_013*, *Butyricicoccus*, and *Ruminiclostridium_5* were found in the PC and FP group (Figure 2). Partial microbial community clusters were overlapped between the NC and PC groups, such as genera *Romboutsia*, *Ruminococcus_torques_group*, and *Oscillibacter* (Figure 2). The overlaps in microbial community clusters were found between the NC and FP groups, such as genera *Eisenbergiella*, *Lactobacillus*, and *Clostridiales_vadinBB60_group_ unclassified*.

A comparative microbial function within the cecal digesta of broilers is presented in Table 6. The results demonstrate that cellular community–prokaryotes function was promoted in the PC group compared with the NC group (*p* = 0.006). Fermented product supplementation decreased cellular community–prokaryotes function compared with the PC group (*p* = 0.05).

### 3.4. Correlation between the Abundance of the Genera, Growth Performance, and Cecal Short-Chain Fatty Acid Levels

**Figure 3:**
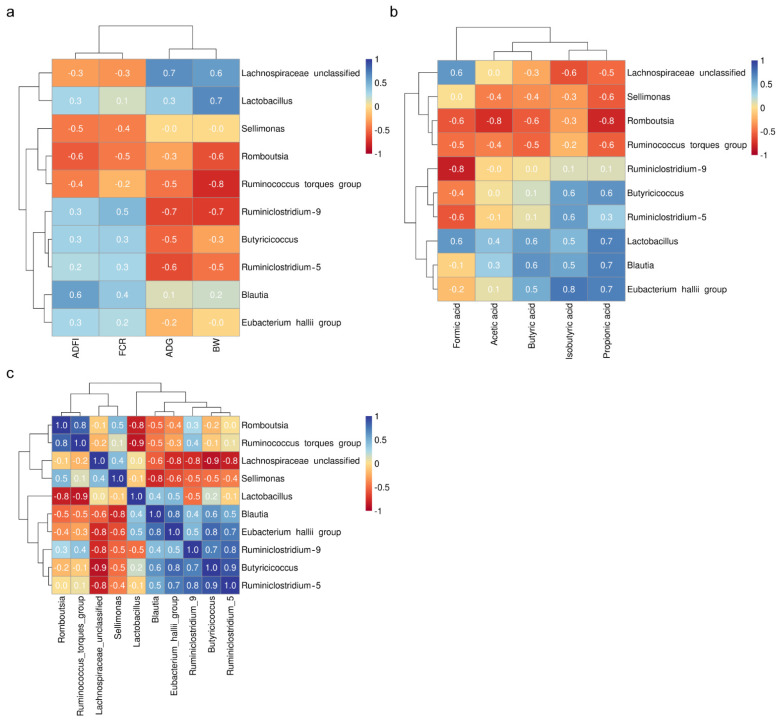
Pearson’s correlation analysis. (**a**) The correlation coefficient between the dominant 10 genera and cecal short-chain fatty acid. (**b**) The correlation coefficient between the dominant 10 genera and growth performance of broilers. ADFI: average daily feed intake, FCR: feed conversion ratio, ADG: average daily gain, BW: body weight. (**c**) The correlation coefficient among the dominant 10 genera. The positive correlations are shown in blue, while the negative correlations are shown in red. The color intensity and the size of the circle are proportional to the correlation coefficients. The values from +1 to −1 indicate the strength of the association.

There was a positive correlation between the abundance of the genus *Lachnospiraceae_unclassified* and *Lactobacillus* in the cecal digesta and average daily gain (ADG) and body weight (BW), the genera *Romboutsia*, *Ruminococcus_torques_group*, *Ruminiclostridium_9*, *Butyricicoccus*, and *Ruminiclostridium_5* were negatively associated with the BW and ADG (Figure 3a). The abundance of the genera *Lachnospiraceae_unclassified*, *Sellimonas*, *Romboutsia*, and *Ruminococcus_torques_group* was negatively associated with the average daily feed intake (ADFI), the genera *Lactobacillus*, *Ruminiclostridium_9*, *Butyricicoccus*, *Blautia*, and *Eubacterium_hallii_group* and were positively associated with the ADFI (Figure 3a). A negative correlation between the abundance of the genera *Lachnospiraceae_unclassified*, *Sellimonas*, and *Romboutsia* and feed conversion ratio (FCR) was observed, the genera *Ruminiclostridium_9*, *Butyricicoccus*, *Ruminiclostridium_5*, and *Blautia* were positively correlated with FCR (Figure 3a). There was a negative correlation between the propionic acid and butyric acid levels and the abundance of the genera *Lachnospiraceae_unclassified*, *Sellimonas*, *Romboutsia*, and *Ruminococcus_torques_group*, the abundance of the genera *Lactobacillus*, *Blautia*, and *Eubacterium_hallii_group* were positively correlated with propionic acid and butyric acid levels (Figure 3b). A negative correlation between the isobutyric acid levels and the abundance of the genera *Lachnospiraceae_unclassified*, *Sellimonas*, and *Romboutsia*, the abundance of the genera *Butyricicoccus*, *Ruminiclostridium_5*, *Lactobacillus*, *Blautia*, and *Eubacterium_hallii_group* was positively correlated with isobutyric acid levels (Figure 3b). There was a negative correlation between the acetic acid levels and the abundance of the genera *Sellimonas*, *Romboutsia*, and *Ruminococcus_torques_group*, the abundance of the genera *Lactobacillus* and *Blautia* was positively correlated with isobutyric acid levels (Figure 3b). A negative correlation between the formic acid levels and the abundance of the genera *Romboutsia*, *Ruminococcus_torques_group*, *Ruminiclostridium_9*, *Butyricicoccus*, and *Ruminiclostridium_5*, the abundance of the genera *Lachnospiraceae_unclassified* and *Lactobacillus* was positively correlated with isobutyric acid levels (Figure 3b). The abundance of the genus *Lactobacillus* was strongly negatively associated with the abundance of the genera *Romboutsia* and *Ruminococcus_torques_group* (Figure 3c). There was a strong negative correlation between the abundance of the genus *Lachnospiraceae_unclassified* and the abundance of the genera *Blautia*, *Eubacterium_hallii_group*, *Ruminiclostridium_9*, *Butyricicoccus*, and *Ruminiclostridium_5* (Figure 3c). In contrast, the abundance of the genera *Blautia*, *Eubacterium_hallii_group*, *Ruminiclostridium_9*, *Butyricicoccus*, and *Ruminiclostridium_5* was positively correlated with each other (Figure 3c).

## 4. Discussion

The overuse of drugs for coccidiosis in poultry leads to anti-coccidial drug resistance in parasites. Hence, probiotics have been considered as alternative candidates for anti-coccidial drugs. It has been demonstrated that *B. licheniformis* can ameliorate body weight gain, intestinal lesion score, and fecal oocysts in broilers challenged with mixed coccidia infection [12]. Our previous study demonstrated that fermented products produced by *B. licheniformis* containing antimicrobial lipopeptides had similar benefits as antibiotics in the growth performance of broilers [15]. We further confirmed that fermented products produced by *B. licheniformis* exhibited anti-coccidial activity in broilers in the present study. In addition, fermented products could modify the cecal microbial community by increasing the genus *Lactobacillus* abundance and decreasing the genus *Ruminococcus_torques_group* abundance. Similar to a previous study [15], the abundance of the genres *Lactobacillus* and *Ruminococcus_torques_group* were also positively and negatively correlated with the growth performance of broilers, respectively. The main findings of this study suggest that fermented products produced by *B. licheniformis* can normalize *Eimeria* species-induced adverse impacts on average weight gain and cecal microbiota of broilers.

Gut microbial balance plays a critical role in maintaining the health and growth of poultry by modulation of the nutrient digestion, intestinal function, and immune system [20]. The intestinal microbiome can be affected by host and diet and overgrowth of pathogenic bacteria in the gut leads to systemic infection [20]. It has been reported that *E. tenella* infection can cause an intestinal microbial imbalance in broilers by increasing the pathogenic bacteria abundance and decreasing the beneficial bacteria abundance, thereby promoting gut damage [17,18]. The *Ruminococcus torques group* genus is associated with gastrointestinal diseases by the degradation of mucin in the gastrointestinal tract, resulting in facilitating gut dysfunction [21,22].

In broilers, the genus *Ruminococcus torques group* abundance is inversely correlated with the growth performance [15,23]. Thus, the genus *Ruminococcus torques group* can be considered as pathogenic bacteria. In the present study, fermented products produced by *B. licheniformis* can normalize the genus *Ruminococcus torques group* abundance in the cecum of broilers under coccidial challenge. The genus *Ruminococcus torques group* abundance is negatively associated with the growth performance in broilers under coccidial challenge, which is in agreement with previous studies [15,23]. In beneficial bacteria, it has been demonstrated that *Lactobacillus* species are able to inhibit *E. tenella* sporozoite invasion in vitro [24]. *Lactobacillus*-based probiotics also exhibit anti-coccidial properties in broilers [2,25]. In the present study, fermented products can increase the genus *Lactobacillus* abundance in the cecum of broilers exposed to coccidial challenge. Furthermore, the genus *Lactobacillus* abundance is positively associated with the growth performance in broilers under coccidial challenge, which is also in agreement with previous studies [15,23]. These results imply that fermented products produced by *B. licheniformis* may inhibit *Eimeria* oocyst development in the cecum of broilers by increasing the genus *Lactobacillus* abundance and decreasing the genus *Ruminococcus torques group* abundance. In our study, some bacteria are specifically sensitive to fermented products or coccidial challenge treatment in the cecum of broilers, such as genus *Romboutsia*. It has been reported that the administration of *Lactobacillus* species can decrease the genus *Romboutsia* abundance in the feces of laying hens and also improve the laying rate [26]. In the present study, the genus *Romboutsia* abundance is negatively correlated with the genus *Lactobacillus* abundance in broilers, which is in agreement with a previous study [26].

We also observed that fermented products improve the average daily gain and also decrease the abundance of the genus *Romboutsia* in the cecum. Thus, these findings imply that fermented products specifically attenuate the genus *Romboutsia* abundance and the genus *Romboutsia* may play a significant factor in the growth traits of poultry. In addition to the genus *Romboutsia*, the genera *Lachnospiraceae_unclassified* and *Sellimonas* was specifically decreased in broilers exposed to coccidial challenge (PC and FP group). The genus *Lachnospiraceae_unclassified* may have a beneficial effect on gut development and health by the production of short-chain fatty acids [27]. A recent study has demonstrated that the genus *Sellimonas* is reduced in abundance in the hens challenged with *Salmonella Typhimurium* [28]. However, the abundance of the genera *Lachnospiraceae_unclassified* and *Sellimonas* in the cecum of broilers were not affected by fermented products in our study. Therefore, the role of genera *Lachnospiraceae_unclassified* and *Sellimonas* in the cecum of broilers still need to be confirmed. Taken together, these results suggest that fermented products increase certain beneficial bacteria populations and reduce the pathogenic bacteria populations in the gut of broilers. The modification of gut microbiota by fermented products can help to prevent coccidiosis in broilers.

In the cecum, the short-chain fatty acid and microbial community exert an important role in maintaining gut health and promoting growth in broilers by regulating the intestinal morphology and immune response [29]. Short-chain fatty acids and microbial communities interact with each other via a complicated mechanism in order to create a beneficial environment for the growth of broilers [30]. In this study, some bacteria (*Sellimonas*, *Romboutsia*, and *Ruminococcus torques group*) in the cecal digesta were inversely correlated with short-chain fatty acid levels, indicating that these short-chain fatty acids in the cecum might inhibit these bacteria growth. These bacteria were also negatively associated with the growth performance (BW, ADG, and ADFI), implying that these bacteria might be unfavorable to gut health. The genus *Lactobacillus* abundance was positively associated with the short-chain fatty acid levels in the cecum and growth performance (BW and ADG), indicating *Lactobacillus* could prevent coccidiosis and improve growth by production of short-chain fatty acids. A strongly negative correlation between the genre *Lactobacillus* and *Ruminococcus torques group* was also observed in the present study. The administration of *Lactobacillus* in the diet can improve intestinal health and reduce the mortality of broilers suffering from necrotic enteritis [31]. To sum up, *Lactobacillus* may inhibit the growth of harmful microbes in the cecum by the production of short-chain fatty acids, thereby improving the gut health and growth in broilers under coccidial challenge.

The antimicrobial mechanisms of antimicrobial lipopeptide have been widely proposed [32]. Previous studies have reported that *B. licheniformis* can synthesize antimicrobial lipopeptides [33,34]. Surfactin, one of *B. licheniformis*-derived antimicrobial lipopeptides, exhibits antibacterial activity against a wide range of Gram-positive bacteria, such as *Listeria monocytogenes* and Methicillin-resistant *S. aureus*, but does not cause hemolysis [35,36]. Our previous findings have demonstrated that surfactin isolated from fermented products inhibits the growth of *C. perfringens* and *B. hyodysenteriae* [13,14]. In addition to bacteria, surfactin also exhibits anti-parasitic activity against *Nosema ceranae* and *Plasmodium falciparum* [37,38]. Surfactin can reduce parasitosis development of *N. ceranae* by direct exposure to spores of *N. ceranae*, resulting in a reduction in infectivity [38] Surfactin is also an inhibitor of intraerythrocytic growth of *P. falciparum* through inhibition of NAD+ and acetylated peptide [37].

Our preliminary results (data not shown) have demonstrated that surfactin purified from *B. licheniformis*-fermented products inhibits sporulation of the *Eimeria* oocyst and promotes the death of merozoite in vitro, implying that surfactin may attack *Eimeria* species directly. In addition to antimicrobial activity, surfactin also exhibits an inhibitory effect on lipopolysaccharides-induced inflammation in vitro [39]. Moreover, *B. licheniformis* also normalizes the gut microbiota, thereby creating a healthy gut environment by competitive exclusion of pathogens for the prevention of *Eimeria* species infection. Thus, the potential anti-coccidial mechanisms we proposed are (1) surfactin inhibits *Eimeria* oocyst growth in the gut, (2) surfactin promotes immunomodulation in the gut mucosal immune system, and (3) *B. licheniformis* regulates microbial community by competitive exclusion of pathogens or production of antimicrobial lipopeptides. However, the precise mechanism of how fermented products exert anti-coccidial activity in the prevention of coccidiosis remains to be investigated.

Although the diets were formulated to meet or exceed the requirements of Ross 308 broiler, broilers were kept in the cage in the present study and body weight was less than expected at 35 days of age (–7.6%) compared with the Ross 308 broiler management guide 2019 [40]. Aviagen management handbook is mainly designed to optimize the growth of Ross 308 broiler for commercial purposes using a floor litter rearing system with advanced environmental control. Previous studies have reported that the body weight of broilers from the floor litter group is heavier (+9.6 to 12.0%) than those from the cage group [41,42,43]. Therefore, the difference between rearing systems is a possible reason why the lower body weight of broilers was observed in the present study. It has been demonstrated that coccidial challenge without vaccination has lower body weight and higher feed conversion ratio in broilers [44]. In our study, the body weight (35 days of age), average daily gain (day 21 to 35 and day 1 to 35 of age), and feed conversion ratio (day 21 to 35 and day 1 to 35 of age) are worsened in the PC group, which is in agreement with a previous study [44]. Fermented product supplementation could normalize the body weight (35 days of age) and average daily gain (day 21 to 35 and day 1 to 35 of age) in broilers exposed to coccidial challenge. However, the feed conversion ratio of the FP group was not improved at 21 to 35 days and 1 to 35 days of age since the average daily feed intake was increased. The increased feed intake was also observed in the PC group. There is no clear explanation for the increased feed intake in the PC and FP group, but we can speculate that the nutrient digestion and absorption in the PC group is severely impaired due to coccidial challenge compared with the FP group. It has been reported that the blood glucose levels of broilers under coccidial challenge are decreased, and blood glucose levels are negatively associated with appetite [45,46]. Thus, the blood level in the PC group is supposedly lower due to the low efficiency of nutrient absorption, thereby stimulating appetite in the hypothalamus. fermented products may promote nutrient utilization by the production of digestive enzymes, resulting in an increased weight gain. In addition, it has been reported that probiotics (such as *Lactobacillus*) can stimulate appetite by activation of ghrelin receptor (hunger signal) in the hypothalamus [47]. Therefore, the increased feed intake in the PC and FP groups may be regulated by a different mechanism.

The cellular community–prokaryotes of KEGG function includes quorum sensing and biofilm formation [48]. Bacteria can regulate the virulence factor production by quorum sensing and virulence factors increase pathogen colonization, immunoevasion, and immunosuppression [48]. The bacterial biofilm formation promotes pathogen adhesion to the surface of the gut and it also associates with antimicrobial resistance [49]. In our study, the cellular community–prokaryotes function was up-regulated in the PC group, indicating that *Eimeria* species may stimulate pathogen colonization in the gut and suppress host immune response. However, the effect of cellular community–prokaryotes function in the cecum of broilers was decreased by fermented products in broilers exposed to coccidial challenge. The results also support the potential mechanism that fermented product supplementation may create a healthy gut environment by competitive exclusion of pathogens. Furthermore, the anti-coccidial index evaluated by relative body weight gain, survival rate, lesion score index, and oocyst count index was worsened in the PC group, whereas fermented products produced by *B. licheniformis* could increase the anti-coccidial index of broilers under coccidial challenge. This finding is in agreement with the results of Chaudhari et al. [12], who observed that *B. licheniformis* can improve body weight gain, intestinal lesion score, and fecal oocysts in broilers challenged with mixed coccidia infection. Taken together, fermented products produced by *B. licheniformis* can regulate microbial community by competitive exclusion of pathogens for the prevention of *Eimeria* species infection in broilers.

## 5. Conclusions

In conclusion, fermented products potentially improved average daily gain at 21 to 35 days of age and exhibited anti-coccidial activity in broilers. A distinct separation of cecal microbial communities was found between the FP and PC groups. Therefore, fermented products produced by *B. licheniformis* have the potential for development as an anti-coccidial feed additive for broilers.

## Figures and Tables

**Table 1:** Ingredients and nutrition composition of basal diets.

Item	1–20 Day	21–35 Day
Ingredient (%, as fed)		
Corn, yellow	55.42	60.73
Soybean meal	35.52	31.53
Fish meal	3.99	3.63
Vegetable oil	3.52	3.02
Limestone	1.52	1.27
Salt	0.30	0.30
Monocalcium phosphate	0.92	0.78
Vitamin premix ^1^	0.20	0.20
Mineral premix ^2^	0.20	0.20
DL-methionine	0.20	0.20
L-lysine	0.10	0.06
Choline chloride	0.05	0.05
Nutritional content (%, as fed)		
Dry matter	8.89	8.87
Crude protein	22.16	20.63
Analyzed calcium	1.02	0.87
Analyzed total phosphorus	0.69	0.63
Lysine	1.12	0.95
Methionine + Cystine	0.85	0.76
Metabolizable energy (kcal/kg)	3081.1	3057.2

^1^ Premix supplied per kg of diet: 160 mg of choline chloride, 20 mg of D-biotin, 10 mg of nicotine amid, 8.3 mg of α-tocopheryl acetate, 8 mg of cyanocobalamin, 2.2 mg of menadione, 2 mg of pyridoxine HCl, 1.8 mg of all-trans-retinyl acetate, 0.3 mg of folic acid, and 0.02 mg of cholecalciferol; ^2^ Premix supplied per kg of diet: 32 mg of Mn (MnSO_4_·H_2_O), 24 mg of Zn (ZnO), 16 mg of Fe (FeSO_4_·7H_2_O), 2 mg of Cu (CuSO_4_·5H_2_O), 800 μg of I (KI), 200 μg of Co (CoSO_4_), and 60 μg of Se.

**Table 2:** Effect of fermented products produced by *B. licheniformis* on the growth performance and anti-coccidial index of broilers under coccidial challenge.

Item	NC ^1^	PC	FP	SEM	*p* Value		
					NC vs. PC	PC vs. FP	FP vs. NC
Body weight (g/bird)							
1 day	43.8	43.8	44.1	0.07	0.889	0.133	0.101
20 day	823.1	795.3	792.3	14.37	0.380	0.952	0.068
35 day	2065.8	1880.8	2006.7	32.78	0.031	0.144	0.310
Average daily gain (g/d/bird)							
1–20 day	39.0	37.6	35.8	0.68	0.380	0.319	0.067
21–35 day	88.8	77.5	89.0	2.17	0.037	0.050	0.939
1–35 day	57.8	52.5	56.1	0.94	0.031	0.145	0.307
Average daily feed intake (g/d/bird)							
1–20 day	66.0	67.0	69.6	0.99	0.490	0.386	0.208
21–35 day	137.3	165.7	172.8	5.38	0.012	0.566	0.003
1–35 day	95.5	106.5	110.9	2.49	<0.001	0.464	0.006
Feed conversion ratio							
1–20 day	1.7	1.8	2.0	0.06	0.326	0.280	0.074
21–35 day	1.6	2.2	2.0	0.10	0.014	0.384	0.023
1–35 day	1.6	2.0	2.0	0.07	0.011	0.745	0.020
Anti-coccidial index	172.5	112.0	169.9	7.07	<0.001	<0.001	0.581

^1^ NC = Basal diet; PC = Basal diet in combination with the coccidial challenge; FP = Basal diet plus 1 g/kg of *B. licheniformis*-fermented products in combination with the coccidial challenge.

**Table 3:** Effect of fermented products produced by *B. licheniformis* on the cecal short-chain fatty acid levels of broilers in response to the coccidial challenge.

Item	NC ^1^	PC	FP	SEM	*p* Value		
					NC vs. PC	PC vs. FP	FP vs. NC
Formic acid (μM)	1.2	0.7	1.0	0.10	0.065	0.104	0.360
Acetic acid (μM)	60.3	59.6	69.1	4.76	0.961	0.459	0.537
Propionic acid (μM)	1.4	1.9	3.9	0.44	0.481	<0.001	0.020
Butyric acid (μM)	7.8	8.6	12.7	1.02	0.621	0.153	0.064
Isobutyric acid (μM)	0.2	0.4	0.3	0.05	0.366	0.822	0.084

^1^ NC = Basal diet; PC = Basal diet in combination with the coccidial challenge; FP = Basal diet plus 1 g/kg of *B. licheniformis*-fermented products in combination with the coccidial challenge.

**Table 4:** Effect of fermented products produced by *B. licheniformis* on the bacterial species richness and evenness in the cecal digesta of broilers.

Item	NC ^1^	PC	FP	SEM	*p* Value		
					NC vs. PC	PC vs. FP	FP vs. NC
Chao1	63.0	73.7	74.3	2.19	0.016	0.871	0.033
Fisher alpha	7.1	8.4	8.5	0.29	0.031	0.879	0.045
Shannon	2.6	3.0	3.4	0.12	0.004	0.151	0.016
Enspie	3.0	4.0	4.8	0.33	0.002	0.324	0.061

^1^ NC = Basal diet; PC = Basal diet in combination with the coccidial challenge; FP = Basal diet plus 1 g/kg of *B. licheniformis*-fermented products in combination with the coccidial challenge.

**Table 5:** Bacterial taxonomic assignment and ranking within the cecal digesta of broilers.

Item	Relative Abundance (%)			SEM	*p* Value		
	NC ^1^	PC	FP		NC vs. PC	PC vs. FP	FP vs. NC
Phylum							
*Firmicutes*	98.5	99.4	99.3	0.15	0.015	0.132	0.025
*Proteobacteria*	1.3	0.4	0.6	0.16	0.009	0.049	0.021
*Actinobacteria*	0.1	0.2	0.1	0.02	0.010	0.030	0.408
Genus							
*Lachnospiraceae_unclassified*	53.3	41.5	41.5	2.26	<0.001	1.000	0.032
*Ruminococcus_torques_group*	21.5	27.0	17.2	1.51	0.022	0.003	0.019
*Lactobacillus*	2.2	1.4	8.6	1.30	0.026	0.031	0.045
*Ruminiclostridium_9*	2.6	4.2	3.7	0.24	0.001	0.052	<0.001
*Butyricicoccus*	2.0	3.9	3.8	0.32	<0.001	0.780	<0.001
*Blautia*	0.5	2.5	4.8	0.64	<0.001	0.009	<0.001
*Eubacterium_hallii_group*	0.3	1.1	1.6	0.20	0.016	0.095	0.001
*Ruminiclostridium_5*	1.1	2.1	1.9	0.15	0.004	0.510	0.005
*Sellimonas*	2.7	1.4	1.3	0.25	0.001	0.680	0.015
*Romboutsia*	1.6	1.8	0.5	0.20	0.382	0.003	0.001

^1^ NC = Basal diet; PC = Basal diet in combination with the coccidial challenge; FP = Basal diet plus 1 g/kg of *B. licheniformis*-fermented products in combination with the coccidial challenge.

**Table 6:** Differences in microbial functions within the cecal digesta of broilers based on Kyoto Encyclopedia of Genes and Genomes functional categories.

Item	Relative Abundance (%)			SEM	*p* Value		
	NC ^1^	PC	FP		NC vs. PC	PC vs. FP	FP vs. NC
Membrane transport	3.94	3.71	3.79	0.845	0.918	0.972	0.946
Nucleotide metabolism	3.27	3.31	3.34	0.093	0.880	0.879	0.788
Cell motility	1.66	1.65	1.59	0.023	0.948	0.369	0.256
Signal transduction	1.23	1.39	1.28	0.402	0.872	0.916	0.955
Translation	1.07	1.03	1.09	0.187	0.901	0.890	0.989
Energy metabolism	1.06	1.15	1.12	0.097	0.738	0.914	0.811
Amino acid metabolism	0.98	1.17	1.08	0.062	0.225	0.562	0.533
Carbohydrate metabolism	0.83	0.72	0.80	0.045	0.315	0.471	0.781
Replication and repair	0.78	0.75	0.74	0.073	0.864	0.978	0.842
Metabolism of cofactors and vitamins	0.53	0.48	0.51	0.036	0.575	0.718	0.822
Folding, sorting and degradation	0.47	0.43	0.46	0.053	0.751	0.793	0.949
Cell growth and death	0.46	0.47	0.46	0.124	0.970	0.970	1.000
Glycan biosynthesis and metabolism	0.26	0.21	0.23	0.058	0.738	0.857	0.873
Metabolism of other amino acids	0.25	0.25	0.25	0.023	0.995	0.951	0.947
Environmental adaptation	0.24	0.25	0.23	0.004	0.167	0.082	0.228
Lipid metabolism	0.18	0.15	0.16	0.020	0.661	0.887	0.766
Cellular community—prokaryotes	0.16	0.19	0.17	0.005	0.006	0.050	0.670
Xenobiotics biodegradation and metabolism	0.14	0.16	0.15	0.012	0.513	0.694	0.792
Infectious diseases: Bacterial	0.14	0.14	0.14	0.013	0.936	0.953	0.880
Metabolism of terpenoids and polyketides	0.12	0.11	0.11	0.018	0.828	0.912	0.912
Transcription	0.09	0.07	0.07	0.017	0.663	0.871	0.781
Endocrine system	0.05	0.04	0.04	0.072	0.571	0.889	0.665
Transport and catabolism	0.05	0.04	0.04	0.009	0.780	0.985	0.786
Biosynthesis of other secondary metabolites	0.04	0.03	0.04	0.004	0.609	0.720	0.889
Endocrine and metabolic diseases	0.03	0.03	0.03	0.002	0.858	0.573	0.626

^1^ NC = Basal diet; PC = Basal diet in combination with the coccidial challenge; FP = Basal diet plus 1 g/kg of *B. licheniformis*-fermented products in combination with the coccidial challenge.

## Data Availability

The data presented in this study are available on request from the corresponding author.
